# Draft Genome Sequences of One Marine and One Clinical *Vibrio parahaemolyticus* Strain, Both Isolated in Sweden

**DOI:** 10.1128/genomeA.01196-16

**Published:** 2016-10-27

**Authors:** Betty Collin, Lee J. Pinnell, James J. Tallman, Jeffrey W. Turner

**Affiliations:** a Northwest Fisheries Science Center, National Marine Fisheries Service, National Oceanic and Atmospheric Administration, Seattle, Washington, USA; b Department of Life Sciences, Texas A&M University–Corpus Christi, Corpus Christi, Texas, USA

## Abstract

*Vibrio parahaemolyticus* is the leading bacterial pathogen associated with seafood consumption. Here, we report the draft genome sequences of one marine and one clinical strain, both isolated in Sweden. These sequences will inform future comparative analysis of *V. parahaemolyticus* in northern Europe.

## GENOME ANNOUNCEMENT

*Vibrio parahaemolyticus* is a Gram-negative marine bacterium that may live freely or attached to abiotic or biotic surfaces. The majority of marine strains are apathogenic; however, some strains may cause gastroenteritis, often associated with consumption of raw or undercooked shellfish. Clinical isolates frequently carry virulence-associated genes such as the thermostable direct hemolysin (*tdh*) and *tdh*-related hemolysin (*trh*) (1).

In northern Europe, ocean warming is related to a recent increase in *V. parahaemolyticus* infections (2). Further, travel to and from destinations where *V. parahaemolyticus* is endemic may lead to increased reporting of clinical cases at Swedish wards. And more generally, globalization (e.g., increased travel and commerce) may lead to the introduction of virulent strains in the local environment, made more hospitable by ocean warming.

Given the emerging risk of *V. parahaemolyticus* in northern Europe, we sequenced a marine and a clinical strain isolated in Sweden. The marine strain was isolated from the edible blue mussel, *Mytilus edulis*, collected outside Sven Lovéns Center for Marine Infrastructure on the Swedish west coast. This marine strain lacks the *tdh* and *trh* genes, but it does harbor the σ-VPH hemolysin gene (3). According to an established 7-loci multilocus sequence typing (MLST) scheme (4), this strain has a unique *recA* allele (no. 338) and therefore represents a new sequence type (ST1579). The clinical strain was isolated at the central hospital of Karlskrona from a male patient who recently returned to Sweden from vacation in Thailand. This clinical strain was *tdh^+^* and shared the same MLST (ST3) as the pandemic complex.

Late log-phase cultures were grown overnight in tryptic soy broth at 30°C with shaking (100 rpm) (Becton, Dickinson, Heidelberg, Germany). Genomic DNA was isolated using a ChargeSwitch gDNA mini bacterial kit per the manufacturer’s instructions (Life Technologies, Carlsbad, CA, USA). DNA was quantified using a BioPhotometer D30 (Eppendorf, Hamburg, Germany) and stored at −20°C. Sequencing of the marine (KVp10) and clinical (Klin) isolates was completed using the Illumina MiSeq platform, at 100× and 75× coverage, respectively. Reads (2 × 300 bp) were trimmed using TrimGalore! (http://www.bioinformatics.babraham.ac.uk/projects/trim_galore) to remove adapter sequences and low-quality bases. Overlapping paired reads were merged using FLASH (5). Optimal *k*-mer size was determined using KmerGenie (6), and reads were assembled *de novo* using Velvet (7). Draft genomes were annotated using the NCBI Prokaryotic Genome Annotation Pipeline (8), and preliminary analysis was conducted using the SEED Viewer (http://theSEED.org). Both draft genomes share greater than 97% average nucleotide identity with the closed reference genome *V. parahaemolyticus* RIMD2210633 (9), as determined by JSpecies (10). A more detailed analysis of these draft genomes will be the focus of a future publication.

### Accession number(s).

The whole-genome shotgun projects have been deposited at DDBJ/ENA/GenBank under the accession numbers MBTR00000000 (KVp10) and MDTV00000000 (Klin). Additionally, the sequence and profile definitions for each isolate have been deposited in the *V. parahaemolyticus* MLST database (http://pubmlst.org/vparahaemolyticus).
